# P2P Cloud Manufacturing Based on a Customized Business Model: An Exploratory Study

**DOI:** 10.3390/s23063129

**Published:** 2023-03-15

**Authors:** Dian Huang, Ming Li, Jingfei Fu, Xuefei Ding, Weiping Luo, Xiaobao Zhu

**Affiliations:** School of Information Engineering, Nanchang Hangkong University, Nanchang 330063, China

**Keywords:** personalized business model, P2P cloud manufacturing, reverse engineering, deep learning, 3D reconstruction, 3D printing

## Abstract

To overcome the problems of long production cycle and high cost in the product manufacturing process, a P2P (platform to platform) cloud manufacturing method based on a personalized custom business model has been proposed in this paper by integrating different technologies such as deep learning and additive manufacturing (AM). This paper focuses on the manufacturing process from a photo containing an entity to the production of that entity. Essentially, this is an object-to-object fabrication. Moreover, based on the YOLOv4 algorithm and DVR technology, an object detection extractor and a 3D data generator are constructed, and a case study is carried out for a 3D printing service scenario. The case study selects online sofa photos and real car photos. The recognition rates of sofa and car were 59% and 100%, respectively. Retrograde conversion from 2D data to 3D data takes approximately 60 s. We also carry out personalized transformation design on the generated sofa digital 3D model. The results show that the proposed method has been validated, and three unindividualized models and one individualized design model have been manufactured, and the original shape is basically maintained.

## 1. Introduction

The ever-increasing customization and personalization demands of customers and the ever-shortening product life cycle have brought severe challenges to the manufacturing industry. Ubiquitous connectivity, digitization and sharing provide opportunities for personalized production to meet the burgeoning demand for personalized goods [1]. In the framework for personalized production based on digital twins, blockchain and AM [1], and the consensus-oriented cloud manufacturing framework based on blockchain technology [2], professional designers may be required to design the entire product model, or traditional reverse engineering may be used to obtain the 3D data. These processes have problems such as long cycle and high cost. The development of extremely flexible cloud services [3] and novel artificial intelligence technology allows this to be realized at low cost, in high quality and quickly.

With the improvement of current manufacturing intelligence and productivity, computer-aided design and manufacturing (CAD/CAM) and rapid prototyping (RP) have become hot words in the manufacturing field. Traditionally, the two behaviors were handled separately. However, as customer demands continue to increase, there is a growing trend of combining the two, which leads to concurrent engineering [4]. Manually creating 3D models is time-consuming and expensive. For this reason, techniques for automatically reconstructing 3D objects have been developed. This technique is the process of capturing the shape of an object through surface data sampling and generating a CAD model of the part, known as reverse engineering [5]. Reverse engineering is the process of 3D scanning and data acquisition of the original physical shape, followed by data processing and 3D reconstruction to build a 3D model with the same shape and structure. Then, on the basis of the original shape, to copy or redesign the original shape to achieve innovation. These techniques can be subdivided into active and passive approaches [6]. The drawback of active methods (e.g., structured light, laser scanners, laser range maps and medical MRI) is that the reconstruction process can be a costly project [7]. Hence, the described methods are passive methods, which require less equipment and can be more widely applied. As soon as a CAD model is obtained through reverse engineering, a large amount of information can be exported and some operations can be performed, such as mechanical design, finite element (FEM) mesh generation, command code generation for CNC machines, overall property calculation, tolerance analysis, accessibility analysis, etc. This provides great support for personalization. At present, many methods [8,9,10] for reconstructing 3D objects can recover the 3D model of the object with only a single shot, which enables fast and low-cost acquisition of 3D data in reverse engineering.

AM is also known as layer manufacturing, rapid prototyping or 3D printing [11]. Different from subtractive manufacturing techniques, such as milling and grinding. It manufactures designed parts by removing material. Additive manufacturing describes the manufacturing process of joining materials to create parts from 3D model data, usually layer by layer [12]. The appeal of additive manufacturing to companies and industries is clear, as it has not only revolutionized the way final part shapes are obtained, but also offers a promising way to develop highly customized and personalized products [13]. AM empowers intelligent manufacturing, and on-demand personalized customization becomes a new direction of development [14]. With the gradual emergence of commercial value such as easy molding, personalized customization, and rapid manufacturing, the application scenarios of 3D printing are becoming more and more diverse. At present, 3D printing has been widely used in construction, footwear, industrial design, jewelry, engineering, aerospace, dentistry, automobiles and other fields. Some manufacturers have also begun to use 3D printing to manufacture aircraft seats, car engines, etc. [15]. After the production of products with the help of cutting-edge 3D printing technology, the innovation of the products’ production process has been accelerated, and its appearance, design, and internal functions have also been further improved.

In order to cope with the ever-changing demand for personalized services, high design costs, long product manufacturing life cycle and other issues, a p2p cloud manufacturing method is proposed based on the personalized business model [1] and cloud manufacturing framework [2]. The difference between this study and these manufacturing frameworks is that it pays more attention to the entity-to-entity manufacturing process, which is used to solve the problems brought about by the time and cost of product manufacturing. This paper is a complete and complementary work to these frameworks. Based on the proposed method, long-distance transmission of physical objects can be realized. When customers see the products they want in multimedia such as video, they only need to take a screenshot to quickly generate the corresponding entity. With this method, only one photo is needed to get the entity in the photo. First, the YOLOv4 [16] is employed to detect and identify all objects in the photo. The targets are cropped to generate a new image. Then the differentiable volume rendering (DVR) [10] technology is optimized to restore the 3D model of the object based on the new image. A digital model file is produced. Finally, the obtained 3D data can be customized for customers. The entity is produced with 3D printers.

In this research, we propose and implement a novel P2P reverse manufacturing method that combines deep learning and AM technology. such that the method is compatible with fast, low-cost and personalized customization features. By using YOLOv4, object detection and recognition is realized. The conversion of 2D data to 3D data is realize by DVR technology. The production printing of 3D digital models is done by employing AM technology. A further distinction of our work from the limited existing work is the overall improvement of the scheme for 3D data acquisition during reverse engineering. The method is applied to the P2P printing service scenario, and the feasibility of the method is verified through a case study. The contributions of this paper can be summarized as follows: (1) A P2P cloud manufacturing method based on the personalized business model is proposed, which can support on-demand manufacturing and long-distance transmission. the method is an extended study of [1,2], bringing them closer to reality. This will be a fast, low-cost, and convenient P2P cloud manufacturing method in the future. (2) Add object recognition and extraction to the original 3D reconstruction method to improve the clarity of the 3D digital model. (3) Based on the proposed method, the feasibility of the proposed method is verified by using photos from the Internet and reality to produce small solid models.

The remainder of this paper is organized as follows. Section 2 briefly reviews key relevant research streams in personalized business models across various industries, deep learning-based reconstruction methods, and additive manufacturing. In Section 3, a P2P cloud manufacturing method based on the personalized business model is presented In Section 4, according to the customer-centered production model, the small models of the objects are generated from two aspects of network pictures and real photos to verify the feasibility of the proposed scheme. They are employed to verify the feasibility of the proposed scheme. Section 5 discusses the contributions of this paper as well as future research.

## 2. Literature Review

**Personalized business model:** As early as 20 years ago, various industries had a business paradigm of personalized customization. For example: personalized interactive TV advertising [17], personalized medicine [18,19], and personalized web system frameworks [20]. After the introduction of Industry 4.0, the intelligent manufacturing industry has moved towards personalized customization. Wang et al. [21] propose cloud-based manufacturing of personalized packaging. Egon [22] proposes a management tool to guide business model innovation in the direction of personalized products: the business model radar template of personalized products. Qin et al. [23] proposed the paradigm of large-scale personalized intelligent manufacturing. Zhang et al. [24] propose a flexible intelligent manufacturing system under the large-scale personalized manufacturing mode. Personalized, mass-manufactured models are gradually becoming the production paradigm of our generation. Guo et al. [1] propose a personalized production framework based on digital twins, blockchain, and additive manufacturing in the context of Industry 4.0, providing useful guidance and reference for the personalized production paradigm. Zhu et al. [2] propose a framework for cloud manufacturing by integrating blockchain technology. Inspired by [1,2], this paper proposes A P2P cloud manufacturing method that provides a quick, easy, and low-cost solution to reversely obtain 3D digital models.

**3D Reconstruction:** In computer vision, 3D reconstruction refers to the process of reconstructing 3D information from single-view or multi-view images or video streams. Ref. [25] is the pioneering work of using deep learning for depth map estimation. Eigen et al. divide the network into a global rough estimation and local fine estimation, estimate the depth from coarse to fine, and propose a scale-invariant loss function. For 3D reconstruction of singular or multi-view images with voxels, Choy et al. [26] combined LSTM, if the input is only one image, the input is one, and the output is also a result. If it is multi-view, consider the multi-view as a sequence, input it into LSTM [27], and output multiple results. In summary, a 2D-image-to-3D voxel model mapping is established through the network structure of the Encoder-3DLSTM-Decoder. Its disadvantage is that it needs to consider the voxel resolution, the size of the calculation time and the size of the accuracy. Fan H et al. [28] used a deep network to directly generate a point cloud from a single image, solved the problem of generating 3D geometry based on a single image object, and created a precedent for single-view 3D reconstructed point cloud representation.

Wang N et al. [29] propose an end-to-end neural network and realized the direct generation of 3D information of objects represented by mesh from a single color image, without the need for point clouds, depth or other more informative data. They used graph convolutional neural networks(GCNNs) to represent the 3D mesh information, using the features mentioned from the input image to gradually deform the ellipse to produce the correct geometry, The core idea of this paper is to use an ellipsoid as the initial shape of any object, and then gradually turn this shape into a target object.

For differentiable rendering, Chen et al. [30] propose DIB-Render, through which the gradient can be analyzed and calculated, which can be used to solve the basic rasterization steps of discrete allocation operations, with a non-differentiable rendering pipeline. The key to their approach is to treat rasterization as weighted interpolation, allowing image gradients to be back-propagated through a variety of standard vertex shaders within a single frame, resulting in single-image 3D object prediction and 3D texture object generation, both using specialized 2D supervision for training. Niemeyer M et al. [10] propose a differentiable rendering formulation that can represent continuously 3D information for implicit shape and texture representations. They can learn implicit shape and texture representations directly from single or multiple RGB images without 3D supervision and result in watertight meshes.

**Additive manufacturing:** Additive manufacturing is defined as the process of building 3D objects by joining materials layer by layer [20]. It is one of the most promising methods, which offers clear advantages in reducing material waste, time bottlenecks, and setup costs compared to conventional methods [31]. Due to the advancement of new technologies, the application of additive manufacturing in various industries, such as [32,33,34], is increasing. As a developing technology to manufacture precise and intensified complex objects by increasing production speed, it may offer an alternative to conventional manufacturing techniques in the near future [35]. Compared with traditional building material manufacturing, additive manufacturing can be manufactured according to design [36]. It provides strong support for personalized customization with higher customer participation. The integration of additive and subtractive manufacturing [37,38] has enormous potential to revolutionize how products are designed, manufactured, and delivered to customers in the form of products.

## 3. A Proposed P2p Cloud Manufacturing Method

Personalized production is a promising model towards the pursuit of expressing individual characteristics of human nature. AI and additive manufacturing can truly transform individual needs and preferences into personalized products and services at an affordable cost through ubiquitous connectivity, digitization, and sharing throughout the product lifecycle. In this section, a P2P cloud manufacturing method based on a customized business model is proposed.

**Figure 1:**
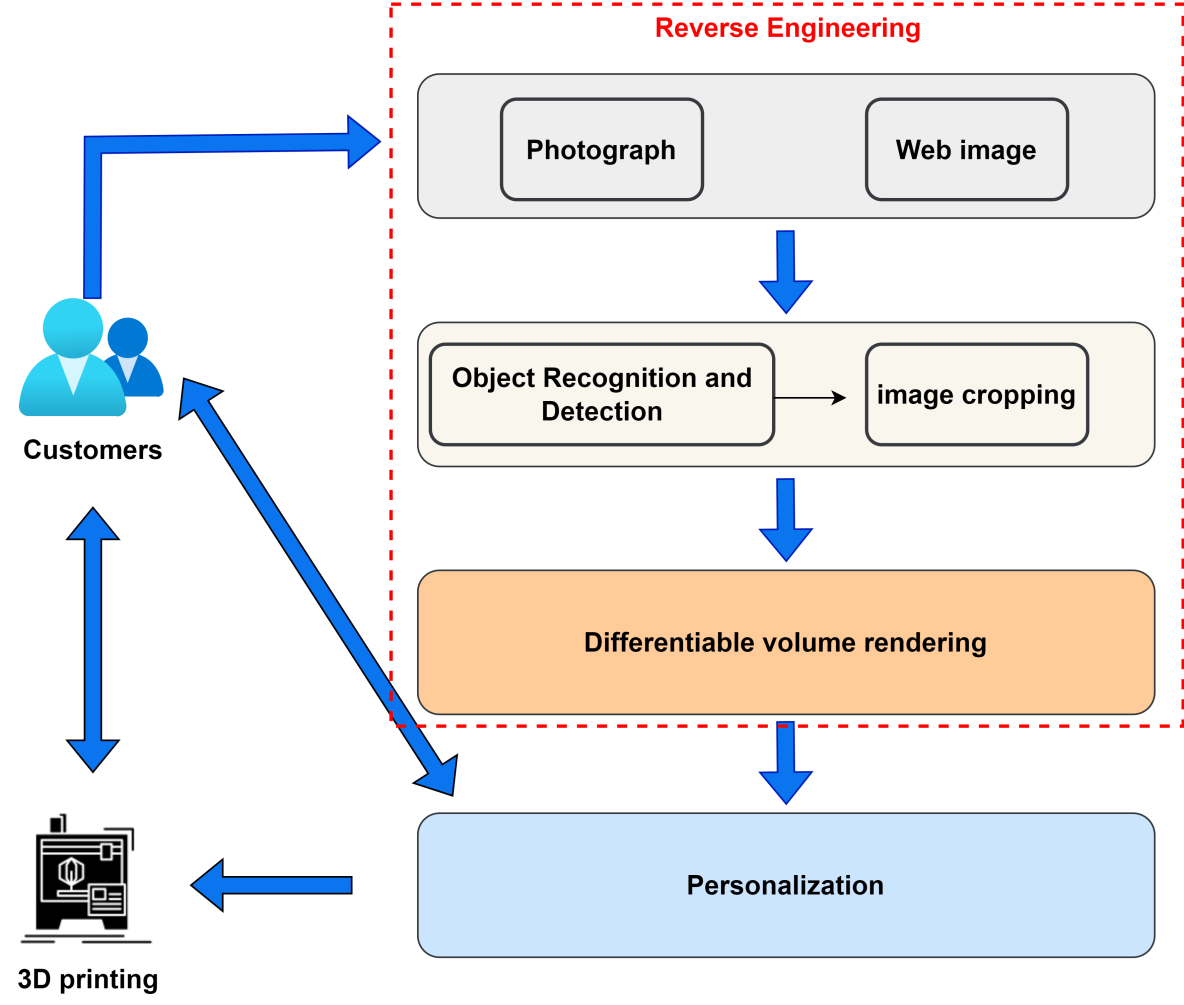
A P2P cloud manufacturing method based on personalized business model.

As shown in Figure 1, customers are involved in the entire product life cycle from design to manufacturing. Customers can take pictures with digital cameras, or download screenshots on fixed and mobile terminals such as tablets and smartphones. This process involves long-distance transmission. AI-powered reverse engineering integrates image preprocessing and single-view reconstruction in the product, linking customer and model production. After the model is produced, the customer participates in the customization process of the model, which is a process of mutual feedback. The printing and production of products is also a process that requires customers and manufacturers to communicate their needs with each other, which is equivalent to the completion of the final product.

Two situations are considered: the object the customer wants is not local; the customer sees the object he wants on the Internet but has no model data. In the first case, simply take a photo of the product remotely. In the second case, just download a screenshot of the product you like. This process is entirely based on images provided by customers based on their needs and preferences. It provides customers with the greatest freedom of choice. Ubiquitous connections and sharing enable long-distance transmission of pictures.

The captured pictures may contain multiple objects, and it is difficult for the current 3D reconstruction technology based on deep learning to reply to the 3D information of each object picture. In view of this, preprocessing of the target image is necessary. YOLOv4 is used for object recognition and detection in pictures. The model output object contains the top, bottom, left, and right coordinates of all detected objects. Since cropping starts at the origin of the original image, the new coordinates are defined as follows:

(1) $$Top_{n}=Max\left(0,top_{r}-4.5\right),$$

(2) $$Left_{n}=Max\left(0,top_{r}-4.5\right),$$

(3) $$Bottom_{n}=Min\left(W,Bottom_{r}+5.5\right),$$

(4) $$Right_{n}=Min\left(L,Right_{r}+5.5\right),$$

where ${\left\{top/Left/Bottom/Right\right\}}_{n}$ denote the new coordinates of the top, left, bottom, and right, respectively. ${\left\{top/Left/Bottom/Right\right\}}_{n}$ denote the top, left, bottom, and right coordinates returned by the YOLOv4 model, respectively. Max() denotes the max function. Min denotes the minimum function. *W* and *L* denote the width and length of the original image, respectively. For better calculation in the neural network, square pictures are required. The Algorithm 1 is as follows:
**Algorithm 1:** Square image generator.1:top, bottom, left, right = 0, 0, 0, 02:fill = round(abs(L − W) /2)3:**if** The length of the original image is greater than or fixed to the width **then**4:   top, bottom = fill, fill5:**else**6:   left, right = fill, fill7:**end if**

Algorithm 1 calculates the part that needs to be filled, which is filled with white. The 3D data of the object can be recovered from this image. A digital model can be obtained simply by determining the shape and texture of the object. DVR technology implicitly represents the shape $f_{\theta }$ and texture $t_{\theta }$ of the 3D model. The gradient from the surface depth is:

(5) $$\frac{\partial \hat{d}}{\partial \theta }={\left(\frac{\partial f_{\theta }\left(\hat{p}\right)}{\partial \hat{p}}\cdot w\right)}^{-1}\frac{\partial f_{\theta }\left(\hat{p}\right)}{\partial \theta },$$

where $f_{\theta }$ denotes the occupancy network [39], which outputs the occupancy probability of each point in the 3D space. θ denotes the network parameter, which only involves computing the gradient at the point $\hat{p}\in R^{3}$. *w* denotes the vector of the camera pointing to a certain pixel point, and its intersection with $f_{\theta }\left(p\right)$ is $\hat{p}$. The input image *i* is encoded using the ResNet18 [40] network $g_{\theta }$:

(6) $$g_{\theta }\left(i\right)=Z,$$

where *Z* is a latent vector of 256 dimensions. The shape and texture of the 3D model are represented as:

(7) $$f_{\theta }\left(p,z\right)=T,$$

(8) $$t_{\theta }\left(p,z\right)=RGB,$$

where $p\in R^{3}$ denotes a point in space. z∈Z denotes the encoder output vector. 3D surfaces are implicitly determined by the occupancy probability T∈[0,1]. The texture of the object is given by the RGB values on the surface of the object. Five fully connected ResNet blocks and ReLu activation functions are used to implement the combined network. The output dimension of the last layer of the model is 4, one of which is occupancy probability, and the three dimensions are texture.

After reverse engineering the initial 3D model, in order to design a product model for individual needs and preferences, it is necessary to develop an effective information recommendation strategy. Designers integrate customer preferences into product design and continuously communicate with customers. Additive manufacturing also provides designers with many design-assisted design tools. Generative design, for example, is achieved through a combination of topology optimization and additive manufacturing, while optimizing topology and material distribution [41]. A digital model of the product (STL, Gcode, etc.) will be generated prior to additive manufacturing.

A designed 3D digital model is imported into the 3D printer. Many 3D printer manufacturers provide specialized model slicing software, which can adjust the actual size of the model, add suitable support structures, etc., before the model is printed. The printed product can be combined with subtractive manufacturing technology to obtain the final shape of the product. Likewise, the printed product is a personalized entity that interacts with customers.

**Figure 2:**
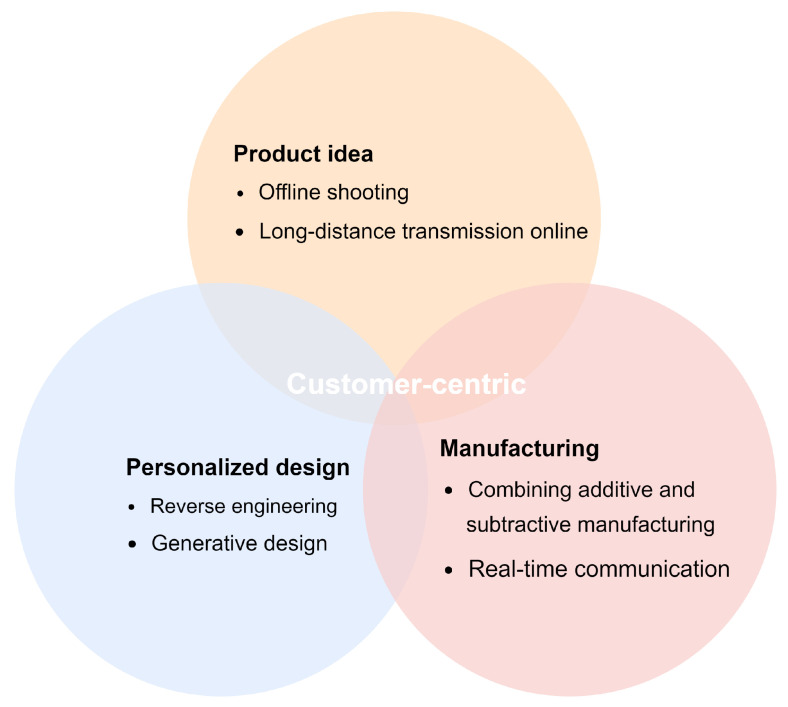
Customer-centric on-demand personalized production model.

Personalized customization is a customer-centric product manufacturing process. Introducing deep learning methods in the reverse engineering stage can reduce costs, shorten design time, and provide customers with long-distance transmission services. The generative design provides designers with more model styles, as well as topology-optimized structures. In the product production stage, additive manufacturing and material manufacturing can be combined. Manufacturers must interact and communicate with customers in real time to ensure product visibility and build connections and trust between customers and manufacturers. The customer-centric customized production model of on-demand manufacturing is shown in Figure 2.

## 4. The Case Studies of the Proposed Cloud Manufacturing Method

Two case studies are utilized to verify the feasibility of the proposed method. Assume that the customer finds the entity he wants while browsing the web or watching a video, but the customer cannot obtain the 3D scanning data of the object, only a screenshot of the website containing the object. Or if the customer sees the object he wants in the real world, he only needs to take a photo containing the object with a digital device to get the object model. The following is to produce the real small objects required by customers from online images and real photos.

### 4.1. Hardware and Software Environment

All procedures are coded in Python 3.8 with Pycharm IDE on a computer of Ubuntu OS with 2.2 GHz Intel i7 CPU, NVIDIA GeForce GTX 1070 GPU, and 16 GB DDR4 RAM. The real-life photos are taken with an iPhone12 with 3.0 GHz CPU, A14 Bionic chip, and 12 million front pixels. The 3D printer model used in the production of the entity is DF3, which is produced by Zhejiang Hangzhou DediBot Intelligent Technology Co., Ltd. [42] in China. Its printing method is FDM (Fused Deposition Modeling), the printing accuracy is 0.1 mm, and the printing speed is 30–100 mm/s. It supports digital model printing such as stl and obj. The specific parameters of the printer are shown in Table 1.

### 4.2. Generating Small Solid Models from the Images

**Figure 3:**
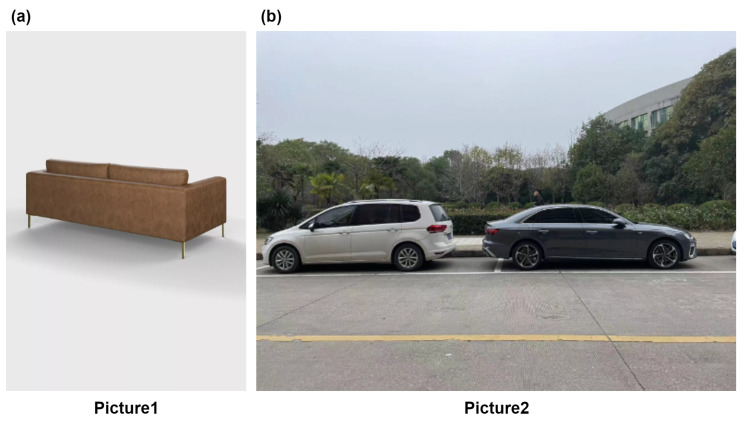
The images used for cloud manufacturing.

**Figure 4:**
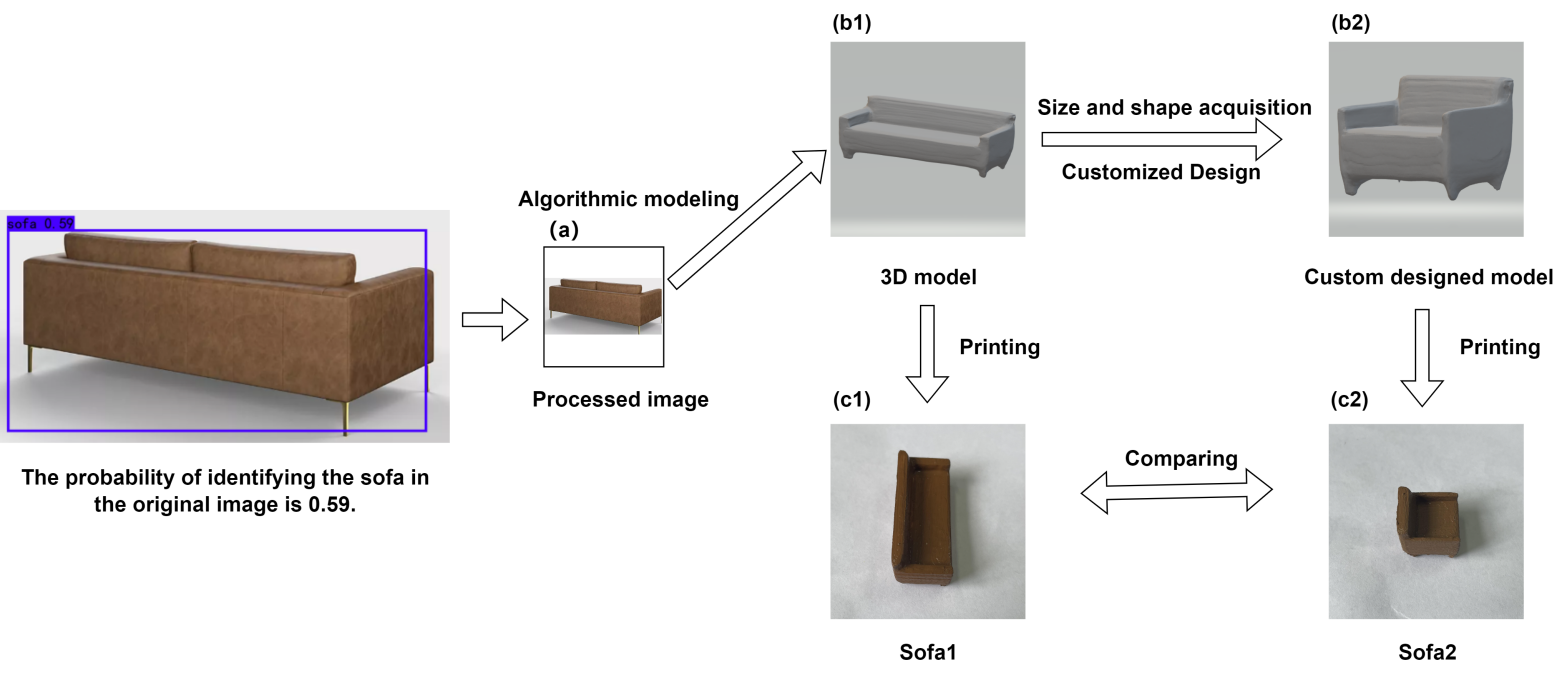
Two small sofa models with different shapes are manufactured using the proposed method.

A picture from a webpage [43] is downloaded with a resolution of 960×1440 and is named Picture1. A photo with a resolution of 4032×3024 is taken by iphone12 in the real world and is named Picture2. Other information of the pictures are shown in Table 2. Picture1 is a four-seater sofa, as shown in Figure 3a, and Picture2 contains two cars of different shapes, as shown in Figure 3b. After detection and identification by the YOLOv4 network, a small sofa is extracted from Picture1. A new 819 pixel × 819 pixel size sofa picture is generated, as shown in Figure 4a. The picture is input into the YOLOv4 network for detection and recognition. The outputs of YOLOv4 are shown in Table 3. The probability of being identified as a sofa in the original image is 59%. The generated new picture is used as the input of the DVR network to construct the 3D model of the modified sofa, and the produced 3D model is shown in Figure 4(b1). Designers get the size and shape of the sofa, as well as personalized custom design. As shown in Figure 4(b2), a four-seater sofa can be turned into a single sofa. This one-seater sofa has the feature of being more portable. Two small sofas of different shapes have been produced. Figure 4(c1) is the sofa without any modification from the original picture Figure 4a, which is longer; Figure 4(c2) is the sofa modified by modeling customization, which is shorter. The printing parameters of the small solid model are shown in Table 4.

**Figure 5:**
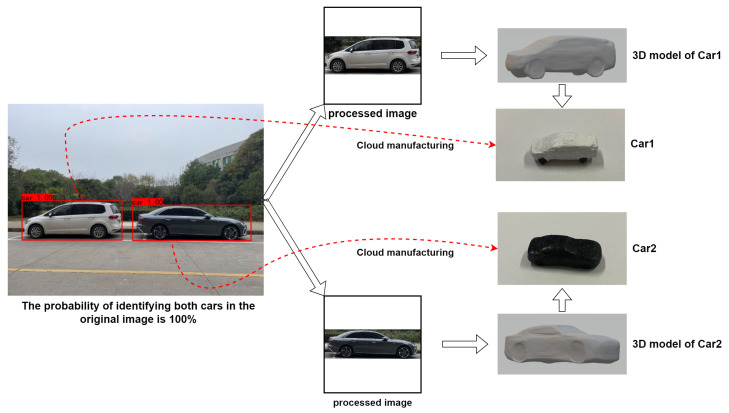
Two small car models with different shapes are manufactured through the proposed method.

Similarly, the Picture2 is input into the YOLOv4 network, and the output results are shown in Table 3. Since two cars were detected in the original picture, two new car pictures are generated. The resolutions of the new pictures are 1591×1591 and 1881×1881 respectively. The new images are fed into the DVR network, which generates 3D models of the two cars. The 3D models are imported into the DF3 printer to produce two small cars. The manufacturing process of the two cars is shown in Figure 5.

**Figure 6:**
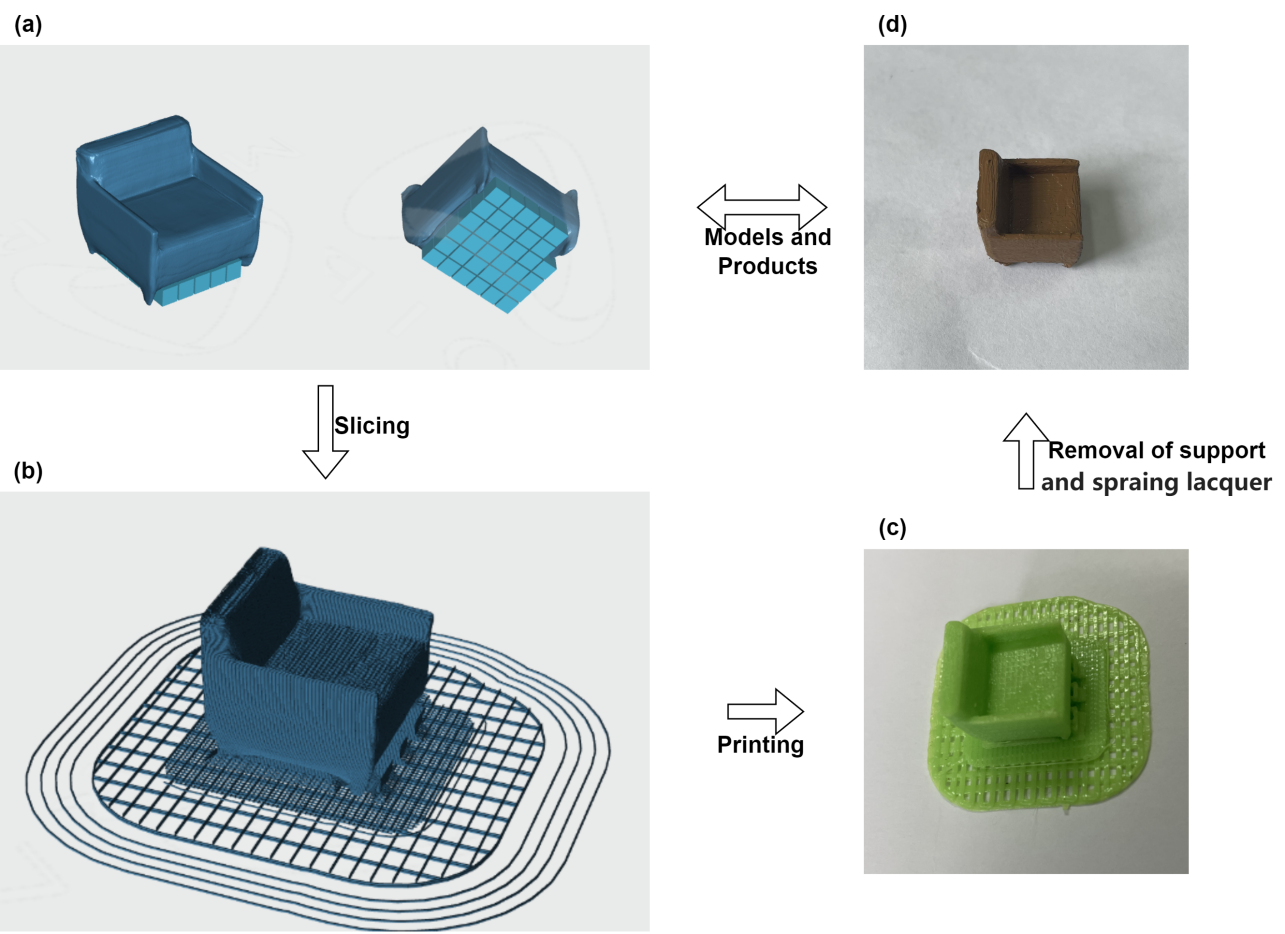
The printing process of Sofa2.

**Figure 7:**
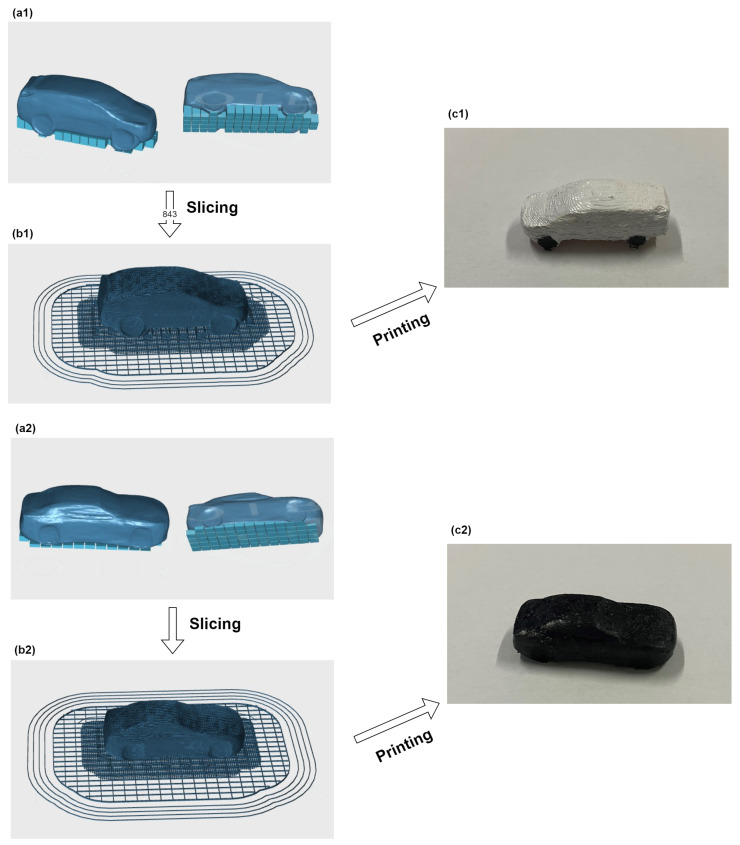
The printing process of two cars. Among them, (**a1**,**a2**) respectively represent the 3D digital models of Picture1 and Picture2 after adding supports, (**b1**,**b2**) represent their sliced models respectively, and (**c1**,**c2**) correspond to the printed small solid models respectively.

MOIRA DF3 is used for printing physical objects. The print samples of the sofa and two cars are showed in Figure 6 and Figure 7. The Sofa2 printing process is taken as an example. The model is imported into a 3D printer. Model b2 in Figure 4 automatically adds supports, see Figure 6a. The model is sliced as shown in Figure 6b. The next step is to print (Figure 6c) and remove the supports (Figure 6d) to form the small sofa. Due to the limitations of current 3D printing technology, the size of the sofa is scaled by 233 times, and the setting is 15.00 mm × 15.89 mm × 13.65 mm. It takes 1.24 h to print the model. Parameters such as printing size and printing time of other models are listed in Table 4. The time to produce a 3D model from a 2D image is shown in Table 5, where Mesh represents the total time used to produce a 3D mesh, and other indicators represent the reconstruction time of each part. It can be seen that it only takes about a minute to recover the 3D structure from a picture. Due to the current limitations of our printer equipment and technology, the small models of sofas and cars are printed, and were not put into actual production. Nevertheless, from these two cases, it can be seen that the sofa and car models have basically been produced. The feasibility of the proposed method is verified.

## 5. Conclusions

To cope with the ever-changing product demand in personalized services, high design costs, long product manufacturing life cycle and other issues, a P2P cloud manufacturing method based on the personalized business model is proposed. This method inherits the on-demand feature of personalized service. Based on the YOLOv4 algorithm and DVR technology, we built an object detection extractor and a 3D data generator, and conducted a case study on a 3D printing service scenario. In the case study, Internet sofa photos and real car photos are selected; the recognition rates of sofa and car are 59% and 100%, respectively. It takes about 60 s to retrogradely convert from 2D data to 3D data. We also carry out a personalized transformation design on the generated digital 3D model of the sofa. Two small sofas and two small car models are printed based on the generated 3D digital models. Judging by the printed results, the proposed method is validated and the prototypes of the sofa and the car were successfully produced. Among them, Sofa2 is transformed from the sofa in the original picture. Sofa1, Car1 and Car2 are all manufactured in their original proportions.

Although the integration of deep learning and additive manufacturing technology overcomes the time and cost problems of traditional reverse manufacturing, more detailed work is required in the future, e.g., applying more powerful printing equipment and technology to realize the value of manufactured products, enriching training data to support the generation of more 3D data to make our method easier to market, and optimizing algorithms to support the generation of objects with more complex structures.

## Figures and Tables

**Table 1:** DF3 printer parameter table.

Parameters	Values
Printer model	MOIRA DF3
Forming size	Φ150× 175 mm
Printer weight	7.2 kg
Printing material	PLA
Printing method	FDM
Printing accuracy	0.1 mm
Printing speed	30–100 mm/s

**Table 2:** The image parameters.

Picture	Resolution	Width	High	Horizontal Resolution	Vertical Resolution	Bit Depth	Size	Inclusions
Picture1	960×1440	960 pixel	1440 pixel	96 dpi	96 dpi	24	238 KB	Sofa
Picture2	4032×3024	4032 pixel	3024 pixel	72 dpi	72 dpi	24	6.51 MB	Cars

**Table 3:** Probability and location of object recognition.

Object	Probability	Top	Bottom	Left	Right
Sofa1	59%	507	48	902	867
Car1	100.00%	1521	216	2153	1807
Car2	100.00%	1591	1958	2164	3840

**Table 4:** Object print parameter settings.

Object	Model Size	Production Time (3D Printing)
Sofa1	15.00 mm × 3.52 mm × 13.65 mm	1.36 h
Sofa2	15.00 mm × 15.89 mm × 13.65 mm	1.24 h
Car1	30.00 mm × 12.45 mm × 12.86 mm	0.31 h
Car2	30.00 mm × 9.80 mm × 11.82 mm	0.26 h

**Table 5:** Time for DVR to produce object 3D model (unit: s).

Object	Mesh	Time (Eval Points)	Time (Marching Cubes)	Time (Refine)	Time (Color)
Sofa1	64.897	10.463	0.993	50.421	2.829
Car1	62.468	8.483	0.989	50.648	2.186
Car2	61.729	8.851	0.991	49.573	2.314

## Data Availability

The data that support the findings of this study are available on request from the corresponding author. The data are not publicly available due to privacy or ethical restrictions.
